# Experimental Incubations Elicit Profound Changes in Community Transcription in OMZ Bacterioplankton

**DOI:** 10.1371/journal.pone.0037118

**Published:** 2012-05-16

**Authors:** Frank J. Stewart, Tage Dalsgaard, Curtis R. Young, Bo Thamdrup, Niels Peter Revsbech, Osvaldo Ulloa, Don E. Canfield, Edward F. DeLong

**Affiliations:** 1 Georgia Institute of Technology, Atlanta, Georgia, United States of America; 2 Department of Bioscience, Aarhus University, Silkeborg, Denmark; 3 Department of Civil and Environmental Engineering, Massachusetts Institute of Technology, Cambridge, Massachusetts, United States of America; 4 Institute of Biology and Nordic Center for Earth Evolution (NordCEE), University of Southern Denmark, Odense, Denmark; 5 Department of Bioscience, Aarhus University, Aarhus, Denmark; 6 Departamento de Oceanografía and Centro de Investigación Oceanográfica en el Pacífico Sur-Oriental, Universidad de Concepción, Concepción, Chile; Universidad Miguel Hernandez, Spain

## Abstract

Sequencing of microbial community RNA (metatranscriptome) is a useful approach for assessing gene expression in microorganisms from the natural environment. This method has revealed transcriptional patterns *in situ*, but can also be used to detect transcriptional cascades in microcosms following experimental perturbation. Unambiguously identifying differential transcription between control and experimental treatments requires constraining effects that are simply due to sampling and bottle enclosure. These effects remain largely uncharacterized for “challenging” microbial samples, such as those from anoxic regions that require special handling to maintain *in situ* conditions. Here, we demonstrate substantial changes in microbial transcription induced by sample collection and incubation in experimental bioreactors. Microbial communities were sampled from the water column of a marine oxygen minimum zone by a pump system that introduced minimal oxygen contamination and subsequently incubated in bioreactors under near *in situ* oxygen and temperature conditions. Relative to the source water, experimental samples became dominated by transcripts suggestive of cell stress, including chaperone, protease, and RNA degradation genes from diverse taxa, with strong representation from SAR11-like alphaproteobacteria. In tandem, transcripts matching facultative anaerobic gammaproteobacteria of the Alteromonadales (e.g., *Colwellia*) increased 4–13 fold up to 43% of coding transcripts, and encoded a diverse gene set suggestive of protein synthesis and cell growth. We interpret these patterns as taxon-specific responses to combined environmental changes in the bioreactors, including shifts in substrate or oxygen availability, and minor temperature and pressure changes during sampling with the pump system. Whether such changes confound analysis of transcriptional patterns may vary based on the design of the experiment, the taxonomic composition of the source community, and on the metabolic linkages between community members. These data highlight the impressive capacity for transcriptional changes within complex microbial communities, underscoring the need for caution when inferring *in situ* metabolism based on transcript abundances in experimental incubations.

## Introduction

Determining how natural microbial communities respond to environmental change is critical for understanding elemental cycling in complex ecosystems. For example, variation in organic substrate and dissolved oxygen (O_2_) availability can dramatically impact microbial metabolism, shifting the rates at which elements are cycled through diverse biochemical pathways [1]–[3]. The patterns of gene and protein expression that underlie microbial responses to environmental change are not well understood in complex natural communities. One approach to characterizing these responses requires experimental incubations of environmental samples under both manipulated and simulated *in situ* (control) conditions. This approach, however, demands a thorough understanding of any potential indirect effects of the experimentation itself that might cause the simulated *in situ* controls to deviate from the actual *in situ* conditions. Such studies can assist in predicting community-level responses to ecosystem change and better define the physical and chemical conditions separating distinct metabolic processes. Here, high throughput sequencing of community RNA (metatranscriptomics) was used to study how experimental incubation (in bottles) under *in situ* conditions affects gene expression in Bacteria and Archaea from a marine oxygen minimum zone (OMZ).

OMZs are critical habitats for exploring how oxygen (or lack thereof) affects the structure and function of natural microbial communities. The largest and most persistent OMZs form in regions of intense upwelling of nutrient-rich water and poor ventilation of intermediate waters, as in the Eastern Tropical South Pacific (ETSP) off the coasts of Chile and Peru [4]–[5]. In these areas, microbial aerobic respiration of sinking organic matter depletes dissolved O_2_, from >200 µM at the surface, to the nanomolar range or lower at the OMZ core [6]. This gradient creates a natural laboratory for studying microbial physiology as community metabolism shifts from aerobic to anaerobic processes. As O_2_ declines, community energy conservation becomes reliant on alternative terminal oxidants, notably oxidized nitrogen compounds (e.g., nitrate). Indeed, denitrification and anaerobic ammonium oxidation with nitrite (anammox) increase with depth into the OMZ, making critical contributions to the flux of N_2_ to the atmosphere and releasing greenhouse gas (nitrous oxide, N_2_O) as an intermediate [7]–[10]. Sulfate may also be an important terminal oxidant in the OMZ water column, where heterotrophic sulfate reduction appears tightly coupled to sulfur oxidation (likely with nitrate) by diverse autotrophic bacteria [11]. While nitrogen and sulfur metabolism, as well as that of other organic and inorganic substrates, is clearly linked to O_2_ availability [12]–[14], the precise oxygen conditions that initiate transitions between aerobic and anaerobic metabolic processes remain poorly defined [1].

Environmental metatranscriptomics, in which high throughput methods are used to randomly sequence the community RNA pool [15], can identify changes in transcription across thousands of genes in response to environmental stimuli, such as O_2_ addition. These methods are increasingly being used in an experimental context, helping to track transcriptional cascades in microbial communities sampled from natural habitats and incubated in microcosms or bioreactors [3], [16]. Such experiments typically manipulate a single environmental variable (e.g., O_2_, light, nutrient concentration), while tightly controlling other variables to mimic conditions *in situ*. However, background changes in the physiology of natural microbial communities in response to sampling or bottle incubation (so called “bottle effects”) can potentially confound simple interpretation of experimental treatments [17]–[18]. Metatranscriptional sampling and bottle effects have rarely been characterized but are potentially critical [19], as they could significantly alter metabolite flux through the community, as well as mask other important transcriptional signals.

In this study, metatranscriptomic sequencing revealed dramatic changes in microbial community gene expression (inferred by proxy from transcript abundance) under experimental conditions. Taxonomically diverse communities from the ETSP OMZ were sampled from an anoxic depth and maintained in shipboard microcosms (bioreactors) with O_2_ concentrations mimicking *in situ* levels. Endpoint samples of community RNA from these bioreactors indicated consistent and significant shifts in protein-coding gene transcription in all treatments relative to *in situ*. These results suggest an overriding transcriptional response to experimental manipulation, with important implications for accurately extrapolating transcriptional patterns in bottle experiments to those in natural communities.

## Materials and Methods

### Ethics Statement

The work described herein was conducted aboard a Chilean Research Vessel via a collaboration with the University of Concepcion, Chile. No specific permits were required for the described field studies, and the collection site is not privately-owned or protected in any way.

### Methods Overview

Shipboard microcosms (bioreactors) were used to monitor changes in microbial community transcription following incubation of OMZ water under *in situ* temperature conditions and without oxygen (O_2_) addition. These bioreactors constitute the “no-amendment control” treatments from two experiments designed to measure the impact of O_2_ concentration on microbial community physiology: Experiment A (O_2_ *gradient*) and B (O_2_ *transition*). These experiments involve multiple bioreactors, each with differing O_2_ concentrations. Comparisons between bioreactor treatments are profiled in a companion paper in which the biogeochemical effects of O_2_ addition are explored in detail (Dalsgaard et al., in prep). Here, we focus specifically on comparisons between the *in situ* microbial communities and the no-amendment *controls*. Each control treatment was replicated in two separate bioreactors per experiment.

### Water sampling

Seawater for bioreactor experiments was collected from the ETSP OMZ off the coast of Iquique, Chile during the Microbial Oceanography of Oxygen Minimum Zones (MOOMZ-III) cruise on the *R/V Vidal Gormaz* (January 8–18, 2010). Water for Experiment A was sampled from 75 m below the surface at Station #3 (20°07′S, 70°23′W; ∼1050 m water depth) on January 12th. Water for Experiment B was sampled from 85 m below the surface at Station #5 (20°06′S, 70°50′W; ∼2800 m water depth) on January 14th. Collection depths at both sites correspond to the upper portion of the OMZ, ∼10–20 m below the oxycline. Water was collected with minimal O_2_ contamination using the pump profiling system described in [11]. The head of the pump cast system was attached to a Seabird CTD equipped with a Seabird SBE 43 oxygen sensor, a fluorometer (Wetlab ECO-AFL), and a STOX oxygen sensor for high-sensitivity oxygen measurements during reactor filling [6], [20].

### Bioreactor filling and seawater incubations

**Figure 1:**
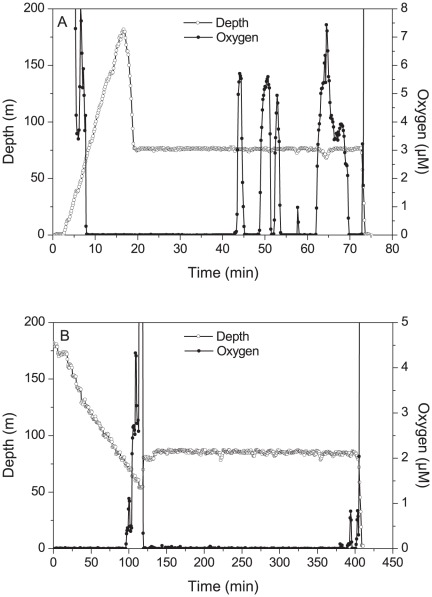
Dissolved O_2_ concentrations (red line, recorded via STOX sensor) during bioreactor filling for Experiment A (top) and B (bottom). Filling began following stabilization of the pump head at a constant depth (green line): at ∼20 minutes in A, ∼125 min in B.

Bioreactors were constructed of 2-L glass cylinders with an inner diameter of 9 cm. A PVC piston with two O-rings was fitted tightly inside the glass cylinder enabling sub-sampling without introduction of a headspace. A 3-mm thick glass plate was glued to the piston limiting contact between PVC and water, thereby minimizing the release of O_2_ to the water. The PVC/glass piston had a hole for the STOX sensor and was penetrated by PEEK tubing for bubbling, amendment (1/16″), and sampling (1/8″). Reactors were rinsed with a brush, acid washed (1 M HCl), and flushed with seawater three times prior to filling. With the piston in place, the inside of the reactor was flushed with N_2_ for 2 min by introducing a piece of tubing through the hole for the STOX sensor. Water was then allowed to flow into the reactor from the pump cast system through a glass valve at the bottom of the reactor, allowing filling from the bottom. N_2_ flow was maintained during filling until water flowed out through the STOX sensor hole. Subsequently, the water was allowed to overflow for three volume changes before the reactor was sealed. Reactors were then transferred to a refrigerated van and placed in a glass aquarium containing water maintained at *in situ* temperature (∼13°C). The van was kept dark, excluding brief periods of illumination under red light. Any dissolved O_2_ that leaked into the reactors during sampling (Figure 1) was purged by bubbling with helium for ∼1.5 hrs. Following purging, the reactors were maintained under the same conditions for ∼30–35 hrs. During purging and throughout the incubation period, O_2_ was monitored continuously via a STOX sensor, with water circulation provided by glass coated magnetic stir bars placed within each reactor.

### Microbial community sampling

Samples of the endpoint microbial community were obtained by filtering the water remaining in each control bioreactor, and its replicate, at the close of the experiment. These samples were compared to samples of the *in situ* microbial community (source water) collected directly from the pump cast system at the time of bioreactor filling. For all samples, the seawater microbial community was filtered through 1.6 µm GF/A prefilters (125 mm dia., Whatman) and then collected onto 0.22 µm Sterivex filters (Millipore) using a peristaltic pump. Sterivex filter units were then filled with RNAlater® (Ambion), capped, flash-frozen in liquid nitrogen, and stored at −80°C until RNA extraction. Less than 20 min elapsed between sample collection (arrival of source water on deck, or the end of each experiment) and fixation in RNAlater®.

### RNA and DNA isolation

Total RNA was extracted from Sterivex filters using a modification of the *mir*Vana™ miRNA Isolation kit (Ambion) protocol used in [21]–[23]. Briefly, filters were thawed on ice, and the RNAlater® surrounding each filter was expelled via syringe and discarded. Cells on the filter were lysed by adding Lysis/Binding and miRNA Homogenate Additive (Ambion) directly to the Sterivex cartridge. Following vortexing and incubation on ice, the lysate was expelled from the filter and further processed via an acid-phenol∶chloroform extraction according to the manufacturer's protocol. The total RNA extract was then incubated with TURBO DNA-free™ to remove genomic DNA, and purified and concentrated using the RNeasy MinElute Cleanup kit (Qiagen).

### rRNA subtraction, RNA amplification and cDNA synthesis

Bacterial, Archaeal, and Eukaryal ribosomal RNA transcripts were removed from total RNA extracts via a subtractive hybridization protocol using sample-specific rRNA probes based on amplicons obtained from PCR of rRNA genes from source water community DNA, as described in [22]. rRNA-depleted total RNA was then amplified using the MessageAmp™ II-Bacteria kit (Ambion) as described previously [15], [21]. Briefly, total RNA was polyadenylated using *Escherichia coli* poly(A) polymerase and converted to double-stranded cDNA via reverse transcription primed with an oligo(dT) primer containing a promoter sequence for T7 RNA polymerase and a recognition site for the restriction enzyme BpmI. cDNA was transcribed *in vitro* (37°C, 12–14 hr) to produce microgram quantities of single-stranded antisense RNA. Antisense RNA (∼5–10 µg aliquot) was then converted to double-stranded cDNA using the SuperScript® III First-Strand Synthesis System (Invitrogen) with priming via random hexamers for first-strand synthesis, and the SuperScript™ Double-Stranded cDNA synthesis kit (Invitrogen) for second-strand synthesis. cDNA was then purified with the QIAquick PCR purification kit (Qiagen), restriction-digested with *BpmI* to remove poly(A) tails, and used directly for pyrosequencing

### Pyrosequencing

Using standard protocols (454 Life Sciences, Roche), poly(A)-removed cDNA was purified via the AMPure® kit (Agencourt®) and used to generate single-stranded DNA libraries for use as template in emulsion PCR. Clonally amplified library fragments were sequenced on a Roche Genome Sequencer FLX instrument using Titanium series chemistry (one full plate run per sample, excluding the control bioreactor replicates, which were sequenced using a half-plate run each). Sequencing yields are shown in Table 1. Pyrosequencing data have been deposited in the NCBI Sequence Read Archive under SRA049608.1.

**Table 1:** Sequencing read numbers.

	Experiment A			Experiment B		
	*in situ*	Control	Control, rep^1^	*in situ*	Control	Control, rep^1^
total reads	1,194,554	838,789	505,110	1,085,724	1,289,435	526,403
rRNA reads^2^	338,311	683,660	41,930	758,937	872,817	60,456
non-rRNA reads^3^	790,456	146,458	451,224	314,940	403,884	440,688
nr reads^4^	330,631	72,534	323,199	102,436	224,937	257,066

1 replicate no-amendment control bioreactors.

2 reads matching (bit score >50) SSU or LSU rRNA sequences via BLASTN.

3 non-rRNA reads; duplicate reads (reads sharing 100% nucleotide identity and length) excluded.

4 reads matching (bit score >50) protein-coding genes in the NCBI-nr database (as of May. 31, 2010).

Pyrosequencing data have been deposited in the NCBI Sequence Read Archive under SRA049608.1.

### Data analysis

Duplicate sequencing reads sharing 100% nucleotide similarity and identical lengths, which may represent artifacts of pyrosequencing, were identified using the clustering program CD-HIT [24] and removed from each dataset as in [22]. Non-duplicate reads matching ribosomal RNA genes were identified by BLASTN comparisons to a database of prokaryotic and eukaryotic small and large subunit rRNA sequences (5 S, 16 S, 18 S, 23 S and 28 S rRNA) compiled from microbial genomes and sequences in the ARB-SILVA databases (www.arb-silva.de/). Reads with significant alignments to rRNA (bit score >50) were removed from further analysis.

Protein-coding gene sequences were identified among the remaining non-duplicate, non-rRNA reads by BLASTX searches against the National Center for Biotechnology Information non-redundant protein database (NCBI-nr, as of May 31^st^, 2010) and the Kyoto Encyclopedia of Genes and Genomes (KEGG, as of May 30^th^, 2010). The top reference gene(s) matching each read (bit score >50) was used to designate each protein-coding read. For reads matching multiple reference genes with equal bit score, each matching reference gene was retained as a top hit, with its representation scaled in proportion to the number of genes sharing an equal bit score. The taxonomic identity of each protein-coding sequence was estimated based on the NCBI annotation of the top matching reference gene identified via BLASTX. The relative abundances of these taxonomic affiliations were tabulated according to the NCBI taxonomy using the program MEGAN [25]. Relative gene abundances are expressed as read counts per gene, normalized as a percentage of the total number of reads matching protein-coding genes in NCBI-nr.

### Statistical analysis of differential expression

**Figure 2:**
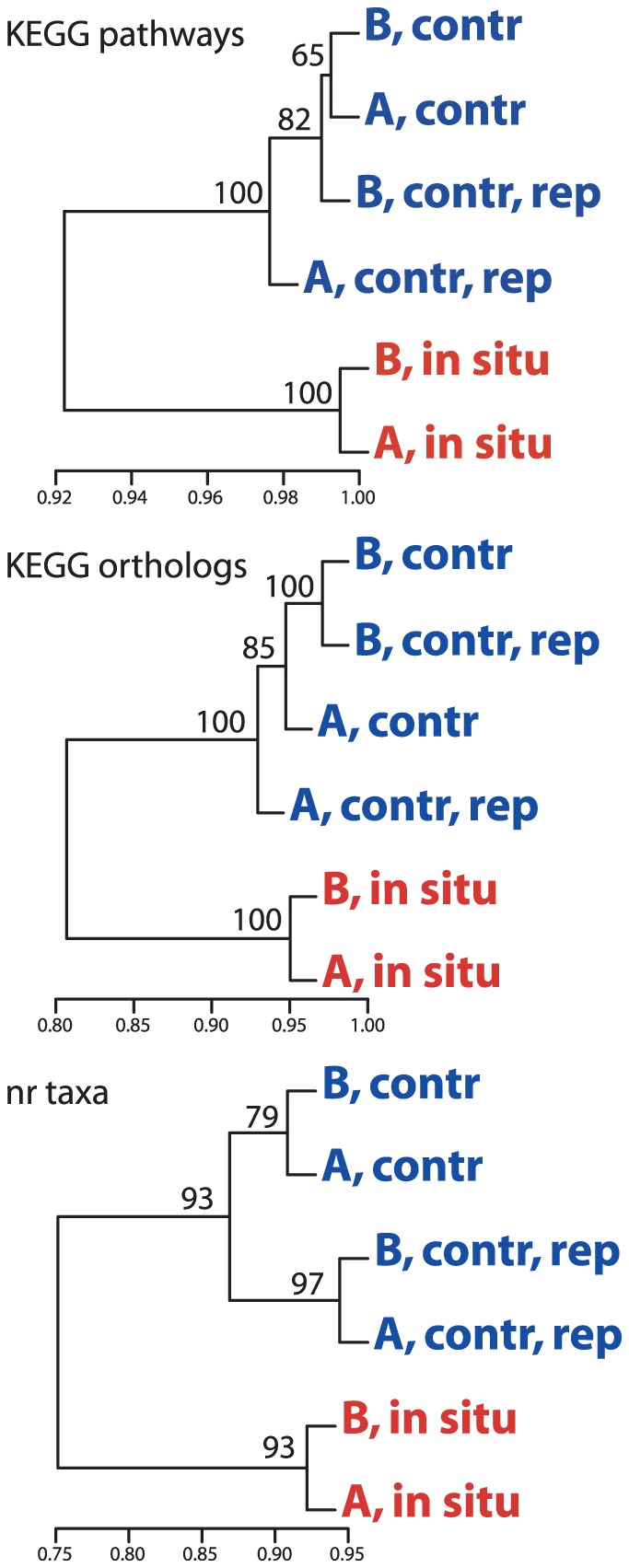
Dataset relatedness based on normalized counts of transcripts matching distinct KEGG Pathways, KEGG orthologs, and NCBI-nr gene taxonomic identifiers. Dendrograms are based on hierarchical clustering of correlation coefficients (X-axis) for arcsine square-root transformed hit counts. Numbers at nodes are probabilities based on multiscale bootstrap resampling (1000 replicates).

Samples were clustered based on the similarity of transcriptome gene content according to the relative abundances of reads matching KEGG Pathways, KEGG Orthologs, and unique nr-taxonomic identifiers (Figure 2). For each sample, hit counts per category were normalized to proportions of total reads matching the KEGG or NCBI-nr database. An arcsine square root transformation was applied to proportions to stabilize variance relative to the mean. Pearson correlation coefficients were calculated for each pair of transformed datasets and used as similarity metrics for hierarchical clustering using the average-linkage method. The robustness of sample clusters was evaluated via multiscale bootstrapping (1000 replicates) using the approximately unbiased (AU) method as implemented using the program pvclust in the R language [26], [27].

The proportion of differentially expressed (DE) genes between samples was estimated via an empirical Bayesian approach in the R program baySeq [28]. Posterior likelihoods of differential expression were calculated for different sets of sample groupings (Models) comparing the *in situ* source water samples to the control replicates (Table 2). As technical replicates were not available for the *in situ* samples, these samples (from the independent experiments) were modeled as biological replicates. The baySeq method assumes a negative binomial distribution of the data with prior distributions derived empirically from the data (100,000 iterations) and dispersion estimated via a quasi-likelihood method. To minimize potential biases owing to small groups of highly expressed genes, count data for baySeq analyses were normalized according to the upper quartile of counts, according to [29].

**Table 2:** Estimated proportion of differentially expressed (DE) KEGG orthologs and genera.

	Est Proportion of DE^1^	
Model	KEGG orthologs	nr genera^2^
(A*_is_*, B*_is_*) (A*_c_*, A*_cr_*, B*_c_*, B*_cr_*)	25.4%	20.0%
(A*_is_*) (A*_c_*, A*_cr_*)	26.4%	10.5%
(B*_is_*) (B_c_, B*_cr_*)	21.3%	14.3%
(A*_c_*, A*_cr_*) (B*_c_*, B*_cr_*)	3.8%	1.3%

1 proportions estimated in baySeq [28] based on posterior likelihoods of DE between sample groups (Model).

2 hit counts per genus inferred from annotations of protein-coding genes identified via BLASTX against NCBI-nr; counts were parsed by taxon according to the NCBI taxonomy in MEGAN.

A and B = experiments A and B, respectively.

*is* = *in situ* sample.

*c* = control sample.

*cr* = control replicate sample.

## Results and Discussion

The experimental incubation of microbial samples under batch growth conditions (e.g., in bottles) has been an important component of microbiological research for decades. Notably, bottle incubations of aquatic samples have provided critical insight into microbial metabolic flux and community composition dynamics in response to environmental perturbation [30]–[34]. Several studies using these techniques have also described “bottle effects” stemming from sampling or confinement in the experimental chamber itself [17], [35]–[36]. Though potentially important, bottle effects are not quantified in most studies, probably because such experiments lack measurements of the *in situ* conditions for comparative purposes, or target a subset of community members that are assumed to be unaffected by bottle enclosure.

Incubations of seawater in microcosms are being increasingly coupled to high-throughput measurements of transcript or protein abundance to study gene expression in microbial communities [3], [16], [37]–[39]. Unbiased interpretation of the results of such studies requires knowledge of community sampling and bottle effects. In the present study, sequencing of total RNA revealed large transcriptional changes in OMZ microbial communities maintained in glass bioreactors. Here, we focus specifically on comparisons between no-amendment (control) treatments and the predominantly anoxic *in situ* microbial community (source water preserved immediately upon collection via a pump cast system), highlighting the potential for substantial transcriptional responses due to experimentation itself.

### Environmental parameters

*In situ* dissolved O_2_ concentrations during source water collection differed between station #3 (Experiment A) and station #5 (Experiment B) (Figure 1). For Experiment A, concentrations hovered near detection limits (<50 nM) during the first half of the bioreactor filling period, but then spiked repeatedly (up to 7 µM) in the second half, reflecting intrusion of O_2_-rich water or depth changes of the pump cast system in the water column due to ship rolling. The control bioreactors were among several reactors filled during this period. Although the exact time at which these bioreactors were filled is not known, the low initial O_2_ concentration in the reactors at the start of the experiment (∼55 nM for the control; Figure S1) suggests that water must have been sampled during the period when O_2_ was at the detection limit. In contrast, water representing the *in situ* sample was collected during the last 15–20 minutes of the sampling period and this sample likely incorporated some of the higher-O_2_ water mass. For Experiment B, O_2_ remained largely undetectable during collection, excluding a brief pulse to 1 µM at the very end of the sampling period (Figure 1).

Despite variability of source water O_2_ concentrations during filling, O_2_ concentrations in control bioreactors during shipboard incubations (measured via STOX sensors within each bioreactor) were similar between experiments and approximated the low concentrations generally observed *in situ*. Dissolved O_2_ concentrations in control reactors averaged ∼50 nM at the start of each experiment, decreased to less than 5 nM following purging with helium gas (∼1.5 hrs), and then gradually increased to <40 nM throughout the duration of the experiment (∼35 hrs total) due to a slight leakage into the reactor or release of O_2_ from the PVC lid and rubber seals (Figures S1 and S2); O_2_ dynamics in the control replicate reactors were similar, with concentrations staying below ∼55 nM (data not shown). These subtle O_2_ changes would go unnoticed using standard oxygen sensors or Winkler titrations, the resolution of which is at least an order of magnitude coarser than that of the STOX sensor [6]. Incubations were run at 13°C and in the dark (excluding brief exposure to red light) to mimic *in situ* conditions at the aphotic depths where the samples were collected.

### Variation between in situ samples

The taxonomic affiliation of protein-coding transcripts, inferred from the annotations of genes recovered as top matches during BLAST searches, was broadly similar between the two *in situ* source water samples, but also varied notably with respect to certain taxa (Figure S3). At both sites, *in situ* transcriptomes contained similarities to transcript datasets recovered previously from the Chilean OMZ [23], being dominated by reads matching Proteobacteria, with particularly strong representation by diverse Gammaproteobacteria, including relatives of sulfur-oxidizing endosymbionts (SUP05 cluster bacterium), and Alphaproteobacteria of the SAR11 clade (*Pelagibacter* sp.). However, other groups varied markedly between the two sites. Notably, transcripts matching genes of the Thaumarchaeal ammonia-oxidizer *Nitrosopumilus maritimus* (Nm; Thaumarchaeota in Figure S3) and close relatives were 9-fold higher in source water for Experiment A (12% of total reads) relative to B (Figure S3). In contrast, transcripts from Planctomycete genes were 55% more abundant in Experiment B (6.5% of total reads) relative to A. In a prior transcriptome study at this site, Planctomycete-like transcripts, including those matching anaerobic anammox bacteria (e.g., *Ca*. Kuenenia stuttgartiensis), were shown to increase in relative abundance along the transition to anoxia at the OMZ core, while Nm-like transcripts were at high abundance in the upper OMZ and oxycline, but were absent in anoxic core samples, presumably reflecting the O_2_ requirement for nitrification [23]. Here, the contrast between the *in situ* samples, interpreted alongside prior patterns, suggests a greater role for aerobic metabolism in Experiment A source water compared to that in B, consistent with the pulses of O_2_-enriched water observed during sampling from the OMZ for Experiment A.

### In situ versus no-amendment control samples – general trends

Differences between *in situ* (source water) and no-amendment control (bioreactor) samples highlight a substantial transcriptional response during incubation. By clustering based on normalized counts of transcripts matching different gene categories, control samples from Experiments A and B were shown (at high bootstrap support) to be more similar to one another, and to their replicates, than either was to its corresponding source water (Figure 2). Indeed, 25% of genes (KEGG orthologs) were estimated to be differentially expressed between source water and the control reactors (all replicates combined; treating the source water samples as biological replicates). In contrast, only 3.8% of orthologs were estimated to be differentially expressed between control reactors from the two independent experiments (Table 2), despite the fact that source water for these two experiments was collected from sites that were separated spatially (∼45 km), sampled on different days, and differed in water mass properties (i.e., O_2_ conditions; see above). Technical replicates of source water transcriptomes at each site were not available, thereby making it challenging to statistically evaluate differential expression proportions between *in situ* transcriptomes. Nonetheless, the convergence of all control reactors on similar transcription profiles, and the clustering of the bioreactor datasets to the exclusion of the source water samples, suggests a shared transcriptional response to sampling and bottle incubation across biological replicates (distinct source water samples), despite attempts to maintain experimental conditions (O_2_, temperature, light) similar to those in the water column.

**Figure 3:**
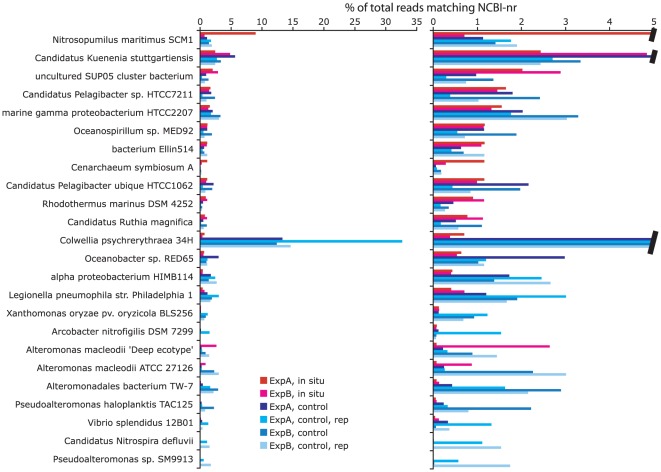
Top 10 most abundant taxa per dataset (*in situ* vs. control), as identified by the NCBI taxonomic affiliation of protein-coding genes matching sequence reads (as top BLASTX hit in searches against the nr database). The right panel shows the data at higher resolution (note change in axis units). Abundances are expressed as percentages of the total number of reads with matches in nr (per dataset). Data are ordered according to increasing rank abundance in the *in situ* dataset for Experiment A.

The broad taxonomic identities of protein-coding transcripts also changed substantially between *in situ* and bioreactor samples (Figures 3 and S3). The taxonomic distribution of the *in situ* community, while variable between source water samples, generally resembled that observed previously for protein-coding sequences from the Chilean OMZ [23]. In contrast, in all bioreactors, experimental incubation elicited a significant increase in the relative abundance of Gammaproteobacteria-like sequences (Figure S3). Specifically, transcripts matching members of the Alteromonadales, a metabolically versatile and readily cultivated Order of Gammaproteobacteria common in marine environments [40]–[41], increased 3.5 to 12.6 fold in relative abundance during incubations, constituting 20 to 43% of all protein-coding sequences in Experiment A and B controls (including replicates). In contrast, transcripts matching most other divisions of the Proteobacteria, notably the Alphaproteobacteria, remained at relatively uniform abundance between *in situ* and control samples (Figure S3). Most other major microbial groups declined in relative abundance during the incubations (Figure S3), likely as a result of the substantial increase in Gammaproteobacteria-like transcripts. These general trends indicate large taxon-specific differences in the physiological response to bioreactor filling and subsequent incubation.

### Genome-specific trends

**Figure 4:**
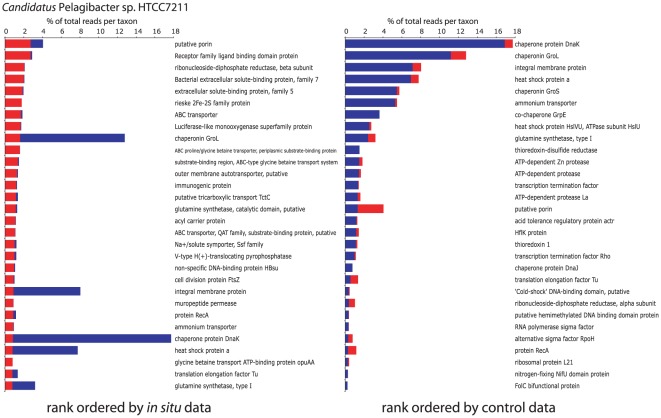
Thirty most abundant genes matching *Candidatus* Pelagibacter sp. HTCC7211 recovered in metatranscriptomes from Experiment B. Abundances are expressed as percentages of the total number of reads with top matches to this genome (identified vi BLASTX against the nr database). Genes are ranked by abundance in the *in situ* (left, red) and control (blue, right) datasets.

**Figure 5:**
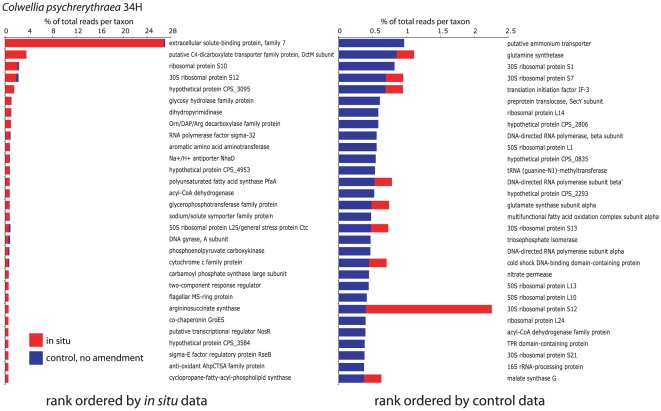
Thirty most abundant genes matching *Colwellia psychrerythraea* 34H recovered in metatranscriptomes from Experiment B. Abundances are expressed as percentages of the total number of reads with top matches to this genome (identified vi BLASTX against the nr database). Genes are ranked by abundance in the *in situ* (left, red) and control (blue, right) datasets.

Examining the functional identities of transcripts matching specific genomes highlighted physiological responses to bottle incubation, although these responses clearly differed among taxa. For example, Figures 4 and 5 show the 30 most abundant protein-coding genes from two genomes identified at high frequency in all experimental bioreactors: the Alphaproteobacterium *Pelagibacter* sp. HTCC7211 and the Alteromonad Gammaproteobacterium *Colwellia psychrerythraea* 34H (data shown are for the Experiment B control). In Experiment B, the abundance of transcripts matching *Pelagibacter* sp. HTCC7211 remained relatively constant between *in situ* and control samples (1.5–2.4% of total reads), although per-gene transcription levels (relative abundance) became much more variable following incubation (stdev of abundance: 1.1 (control bioreactor) versus 0.3 (*in situ*)), suggestive of a greater reliance on a subset of metabolic functions (Figures 3 and 4). In contrast, *C. psychrerythraea* 34H transcripts increased 33-fold in this sample and were much more uniformly distributed across the *C. psychrerythraea genome* during incubation (stdev of abundance: 0.07 (control) versus 1.8 (*in situ*); Figures 3 and 5).

In the *in situ* sample, both *Pelagibacter* and *Colwellia*-like transcriptomes were dominated by diverse genes mediating the detection and transport of organic and inorganic extracellular solutes, including amino acids and cations (e.g., NH_4_^+^). Notably, over 25% of the sequences in the *in situ Colwellia* transcriptome encoded a protein belonging to a family of extracellular solute-binding proteins specific for dicarboxylic and tricarboxylic acids (family 7) [42]. Transcripts encoding a related protein were also abundant in *Pelagibacter*, along with those encoding diverse ATP-binding cassette (ABC) transporters, including those for dipeptides (family 5, solute-binding protein) and the osmoprotectant glycine betaine. This overall strong representation of genes for substrate acquisition is consistent with metaproteomic data showing a prevalence of transport–associated proteins in *Pelagibacter*-like cells in the oligotrophic Sargasso Sea [43]. Our data also suggest strong environmental pressure for nutrient and carbon acquisition by both *Pelagibacter* and *Colwellia*-like heterotrophs in the upper OMZ at the time of sampling.

These taxa showed strong, but distinct, transcriptional responses during enclosure in bioreactors. Whereas the *in situ* transcript pool was dominated by transport functions in both *Colwellia* and *Pelagibacter* (see above), these taxa differed in their functional response to sampling and incubation. The most abundant *Colwellia* transcripts in the control bioreactor encoded diverse proteins mediating transcription and protein synthesis (e.g., RNA polymerase, ribosomal proteins, translation factors) and nitrogen assimilation (e.g., glutamine synthetase, glutamate synthase, ammonium transporter, nitrate permease), indicating a redirection of energy reserves away from substrate acquisition and towards cell growth (Figure 5). In sharp contrast, half of the *Pelagibacter* control transcriptome was represented by sequences encoding chaperones (45%) and proteases (5%), with 35% of all HTCC7211 transcripts encoding the conserved heat shock chaperones DnaK (17%), DnaJ (1%), GroL (11%), and GroS (5%) (Figure 4). In addition to mediating proper protein folding in healthy cells, these chaperones play critical roles in counteracting protein denaturation and misfolding in response to a range of environmental stressors, including extremes in temperature, osmolarity, oxidative damage, pH, UV, substrate availability, and ethanol and heavy metal concentration [44]–[45]. Their overexpression, in combination with that of diverse proteases, suggests a state of cellular stress in *Pelagibacter*-like cells associated with bottle filling and incubations.

The differences in the response of *Pelagibacter*- and *Colwellia*-like cells to bottle enclosure may reflect fundamental differences in how these two clades adapt to fluctuating environmental conditions. *Pelagibacter* species contain some of the smallest genomes of any free-living marine bacteria (∼1.3–1.5 Mbp) and appear specifically adapted for the uptake and assimilation of dissolved organic carbon under limiting nutrient conditions [46]–[47]. Genomic streamlining in these cells has likely helped to ensure a relatively efficient (and uniform) replication rate, but may hinder the ability of these cells to switch metabolic strategies when the environment changes. Indeed, several pathways, such as the canonical glycolytic pathway, are missing key enzymes in *Pelagibacter* [46]. In contrast, *Colwellia*, like other marine heterotrophs, has a genome over three times larger than that of *Pelagibacter* and contains genes for diverse pathways of energy generation and carbon and nutrient acquisition, including glycolysis, the pentose phosphate pathway, fermentation (e.g., via the methylcitrate pathway), and the β-oxidation of fatty acids [48]. *Colwellia* appears capable of utilizing diverse organic molecules for carbon and energy, from simple sugars to amino acids to high-molecular weight compounds such as starch and polyhydroxyalkanoates. Further, *Colwellia* is facultatively anaerobic, having the capacity to conduct complete denitrification to dinitrogen gas under anoxic conditions, but also to use oxygen as a terminal oxidant when available [48]. The metabolic versatility of this bacterium may make it well adapted to exploit changes in substrate availability. Indeed, members of the Altermonadales, including *Colwellia*, appear to be some of the first to increase in cell number following organic substrate enrichment [29], [49]–[51]. Changes in substrate conditions in our bottle experiments may explain the positive transcriptional response observed in these groups.

### Community-level functional trends

**Figure 6:**
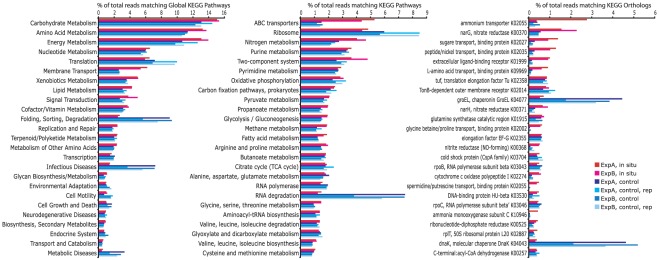
Twenty-five most abundant KEGG categories, ordered according to rank abundance in the Experiment A *in situ* dataset. KEGG categories are shown at increasing levels of resolution: Global Pathways (left), Pathways (center), and Orthologs (right). Abundances are expressed as percentages of the total number of reads with matches in KEGG (per dataset).

The genome-specific patterns described above are reflected in functional trends observed at the community level, without subdivision by taxon. Whole dataset BLASTX searches against the KEGG database confirmed that *in situ* communities from both sites were relatively enriched by transcripts encoding membrane transport functions and environmental signal detection and transmission (e.g., via two-component systems) (Figure 6). Transcripts mediating nitrogen metabolism, notably enzymes of dissimilatory nitrate reduction (e.g., nitrate reductase, *narGH*), were also abundant *in situ*. While these transcripts declined in relative abundance after incubation, likely due to the increase in other functional categories (see below), nitrogen metabolism genes remained among the most highly transcribed in control bioreactors, suggesting a prominent role for nitrogen respiration in experimental incubations (Figure 6 and S4). However, confirming the link between community transcript abundance patterns and metabolic processing (e.g., nitrogen respiration) will require coupling experimental metatranscriptomics analyses to actual biogeochemical rate measurements (Dalsgaard et al., in prep).

In contrast, bioreactor community transcriptomes were enriched in sequences encoding chaperones and protein folding catalysts, RNA degradation proteins, peptidases/proteases, and ribosome related proteins (Figure 6, S4, and S5). In addition, based on BLASTX searches against NCBI-nr, all bioreactors exhibited substantial up-regulation (15–70 fold increase relative to source water) of genes encoding a coat protein (phasin) associated with polyhydroxyalkanoates (*PHA*), a family of polyesters that serve as intracellular carbon and energy reserves during periods of cell stress (Figure S6). This increase is consistent with the high representation of other stress-response genes observed at the community level, as well as in the *Pelagibacter*-like genome (above). Indeed, the vast majority (>95%) of bioreactor phasin transcripts were most closely related to a gene from the Alphaproteobacterium HIMB114 (ZP_06055224.1), a close relative of *Pelagibacter* in the SAR11 clade. These data suggest a potentially important role for PHA storage in these ubiquitous bacteria under stressful environmental conditions. Alternatively, phasin-like proteins may play different yet-uncharacterized functional roles in these pelagic marine microbes.

### Potential Sampling Effects and Implications

Our data from the OMZ confirm that sampling and incubation in experimental chambers can profoundly change microbial community transcription in water collected from anoxic depths, even within “control” treatments where *in situ* conditions are closely mimicked. These results are timely as the increasing application of metatranscriptomic analysis to bottle experiments underscores the need to understand gene expression in response to bottle enclosure. For example, Rinta-Kanto et al. [38] sequenced the endpoint metatranscriptomes of aerobic microbial communities isolated from the Gulf of Mexico (GOM) and incubated (5-days) in microcosms with and without nutrient enrichment. This study did not include comparisons to the *in situ* microbial community, so the potential transcriptional response due directly to bottle enclosure could not be evaluated. However, broad patterns observed in their experiments were similar to those reported for our OMZ incubations. For example, both OMZ and GOM incubations contained high numbers of transcripts encoding proteases and molecular chaperones (e.g., GroEL was the 2^nd^ most abundant gene in the GOM control transcriptomes). Also, the Altermonadaceae, whose transcripts dominated our OMZ control samples, were proportionately enriched in the GOM transcript pool compared to the rRNA gene pool, identifying the potential for this group to contribute substantially to gene expression despite relatively low cell abundance.

In a related study using experimental metatranscriptomics, McCarren et al. [3] observed how activity in a subset of taxa can trigger cascading metabolic responses in other community members. In this analysis, seawater incubations in 20-L carboys were coupled with metatranscriptome sampling to track gene expression at multiple time points following dissolved organic matter (DOM) addition. This study examined bacterioplankton from the oxygen-rich surface layers of the oligotrophic North Pacific gyre (Station ALOHA, Hawaii Ocean Times-series) where community composition and environmental conditions (e.g., oxygen and nutrient availability) differ dramatically from those at the Chilean OMZ sites [23], [52]. Nonetheless, as in our experiments, the relative abundance of transcripts matching coding genes from the Altermonadaceae and other Gammaproteobacteria increased in control bottles (no DOM) during their experiment (27 hrs), effectively doubling to constitute ∼20% of all identifiable protein coding transcripts at the endpoint. Interestingly, the abundance of these groups in the ribosomal RNA gene sequence pool (DNA) remained unchanged or decreased slightly during the incubation, again suggesting that these taxa contribute disproportionately to the total transcript pool during bottle enclosure. Similar to patterns observed in our OMZ incubations, transcripts encoding two-component system proteins and ABC and ion-coupled transporters significantly decreased in abundance in their control bottles, whereas those matching ribosome-related proteins increased. Though other metatranscriptome studies have not directly analyzed bottle effects, several trends from these studies, interpreted alongside patterns from our analysis, suggest that the transcriptional response to bottle enclosure may be similar across diverse experiments and under varying oxygen conditions.

The root causes of bottle effects remain ambiguous. Prior studies have suggested diverse contributing factors, including changes in substrate availability due to adsorption of cells, carbon, or nutrients on glass surfaces, as well as unintended changes in turbulence patterns, chemistry, or trophic dynamics [53]–[57]. Here, bottle effects were similar across all experimental incubations (including replicates), and included a strong positive response from facultative anaerobes in the Altermonadaceae as well as a generalized stress response from diverse members of the Alphaproteobacteria, notably *Pelagibacter*-like cells. As discussed above, these clades may differ in their ability to respond to changes in substrate (nutrients, carbon) availability [30], [58], suggesting that such changes may have occurred in the bioreactors. Alternatively, substrate conditions may have remained constant, whereas the relative abilities of different community members to compete for these substrates might have changed. This could occur if taxa respond differentially to changes in other variables, such as pH, temperature, or O_2_ concentration. Despite our (largely successful) attempts to sample anoxically and to constrain O_2_ conditions at near *in situ* levels (<30 nM), minor shifts in O_2_ concentration during bioreactor filling may have elicited a transcriptional response from the bottle communities. However, the observation of similar transcriptional trends in studies involving aerobic communities (see above) suggests that the bottle effects in the present study may not be linked exclusively to O_2_ exposure. By sparging with helium to remove O_2_, we may also have shifted internal carbon dioxide concentrations, thereby increasing the pH in the bioreactors and potentially disrupting biochemical pathways [59]. As purging with an inert gas is a routine, and typically necessary, step in bottle experiments measuring anaerobic metabolism, characterization of the potential physiological stresses imposed by this technique should be clarified.

Alternatively, the metatranscriptional patterns observed here may have been a response to a combination of interacting variables. These may have included minor and unavoidable temperature and pressure changes during sampling using the pump cast system. Indeed, sampling from suboxic depths using a pump system similar to that used here was shown to have a pronounced effect on bacterioplankton transcription, potentially due to mechanical stress or pressure changes in the sampling tube [19]. Here, both the “*in situ*” and control samples were collected via the pump-based system. However, stress due to sampling may not have been observable in the *in situ* samples if the transcriptional stress response signal lagged the sampling period (∼20 min, including pumping, filtration, and sample preservation). Teasing apart the complex variables underlying the patterns observed here would require multiple targeted experiments and is beyond the scope of most experimental metatranscriptomics studies due to time and cost limitations. However, such studies would benefit from comparisons between *in situ* and control samples, thereby enabling the relative magnitude of sampling bottle effects to be assessed.

The extent to which bottle effects prevent drawing meaningful inferences from metatranscriptional data will depend largely on the magnitude of the effects, which will vary based on the design of the experiment and the composition of the starting community. Bottle incubations are typically designed to assess variation between treatments differing in the presence, absence, or magnitude of a single variable. Metatranscriptomic analysis within this framework can be tremendously informative if between-treatment variation can be adequately resolved. However, large transcriptional bottle effects can obscure our ability to interpret changes in relative transcript abundance among samples, potentially masking subtle changes in gene expression due to treatment variation and imposing a need for additional sequencing to detect lower-frequency transcripts. Additionally, metabolic processes in natural microbial communities exist within a complex web of interacting pathways and species. As the linkages within this web are poorly defined, it is potentially naïve to assume that bottle effects, even if seemingly exhibited by only a subset of the community, do not have indirect effects on all members. The OMZ microorganisms sampled here inhabit particularly steep and dynamic vertical gradients in inorganic substrate and oxygen availability. Consequently, they may be especially responsive to even slight changes in their environment and therefore prone to bottle effects. Metatranscriptional patterns inferred from bottle incubations of these communities may be unlikely to reflect those in nature, even when experiments are seemingly maintained under *in situ* conditions. However, experimental metatranscriptomic datasets, despite the presence of bottle effects, may be particularly powerful when coupled to biogeochemical rate measurements (Dalsgaard et al., in prep), thereby allowing researchers to link community patterns of transcript abundance to actual changes in metabolic processes.

## Supporting Information

Figure S1 **Dissolved O_2_ concentrations in the no amendment control bioreactor (#7) during Experiment A.** STOX oxygen sensors began recording approximately 3 hours after bioreactor filling. Gassing with helium began at ∼3.7 hours, lasting until ∼5 hrs. The increase in O_2_ around 3.7 hours is due to handling of the reactor when setting up the gassing (creating a headspace in the reactor, connection of tubes, etc.). The O_2_ concentration remained constant at ∼55 nM between the start of recording and the start of gassing. Consequently, it should be safe to assume that this was approximately the level of O_2_ in the reactor for the first 3 hours after filling (indicated by the gray line). The increase in O_2_ from 6–26 hours is due to leakage into the reactor or release of O_2_ from the PVC and rubber. Electronic errors have been removed.
(PNG)

Figure S2 **Dissolved O_2_ concentrations in the no amendment control bioreactor (#5) during Experiment B.** STOX oxygen sensors began recording approximately 1 hour after bioreactor filling. Oxygen decreased from 40.7 nM to 17.5 over the first 1.5 hours ( = 15.5 nM h^−1^), potentially due to respiration. This bioreactor had been in use for several days and the release of O_2_ from PVC and rubber was smaller than in Experiment A above. Gassing with helium started at ∼2.7 hours, which created an increase in O_2_ due to the handling of the reactor. The best estimate of starting O_2_ concentration is 40.7+1.03×15.5 = 56.7 nM, as indicated by the grey line. Electronic errors have been removed.
(PNG)

Figure S3 **Taxonomic composition of protein-coding reads.** Taxonomic identifications are based on annotations of NCBI reference sequences recovered as top matches (above bit score 50) in BLASTX searches. Numbers indicate read counts per taxon, normalized to a standard of 100,000 reads per dataset.
(EPS)

Figure S4 **Top 20 most abundant KEGG orthologs identified per transcriptome.** Different graphs reflect the dataset by which the data are ranked (i.e., in the upper right, the orthologs shown are the top 20 identified within the no amendment control dataset from Experiment A). Data from replicate control bioreactors have been excluded for clarity.
(EPS)

Figure S5 **Relative abundance of chaperone genes (blue) relative to other genes (red) in major taxonomic groups in the Experiment B no amendment control metatranscriptome.** Taxonomic identifications are based on annotations of NCBI reference sequences recovered as top matches (above bit score 50) in BLASTX searches. Relative abundances are reflected by circle area.
(EPS)

Figure S6 **Abundance of reads matching the gene encoding phasin, a protein putatively associated with bacteria-produced polyhydroxyalkanoate (PHA) granules.** The abundance of the primary PHA synthesis gene, PHA synthase, is shown for comparison. Abundances are expressed as percentages of the total number of reads with matches to the nr database (per dataset).
(EPS)
