# Supplementary figures and images for: Experimental Incubations Elicit Profound Changes in Community Transcription in OMZ Bacterioplankton
Source: PLoS One. 2012 May 16;7(5):e37118. doi: 10.1371/journal.pone.0037118 (PMC3353902; doi:10.1371/journal.pone.0037118)

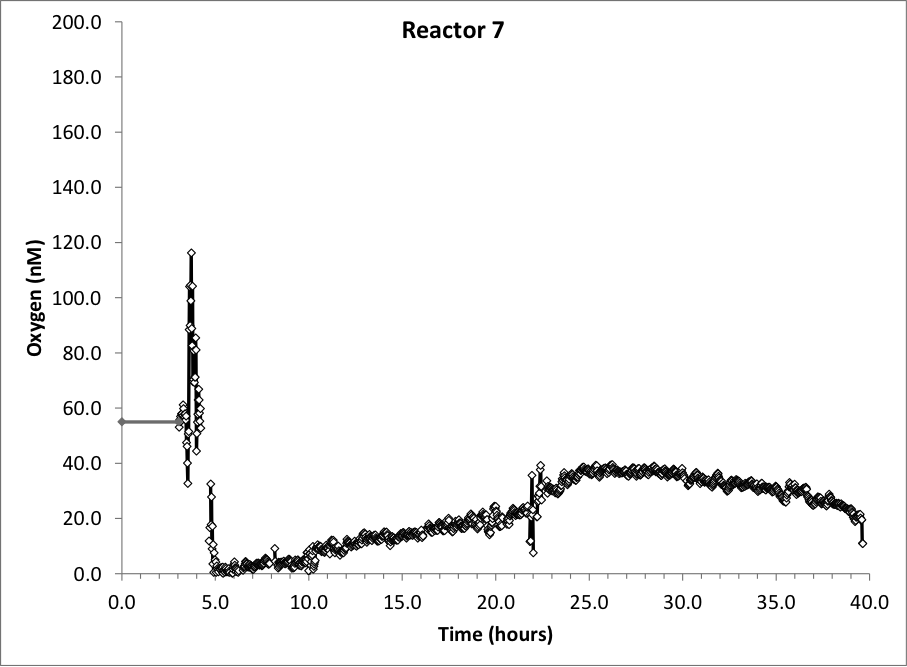

Supplement: Figure S1 — Dissolved O2 concentrations in the no amendment control bioreactor (#7) during Experiment A. STOX oxygen sensors began recording approximately 3 hours after bioreactor filling. Gassing with helium began at ∼3.7 hours, lasting until ∼5 hrs. The increase in O2 around 3.7 hours is due to handling of the reactor when setting up the gassing (creating a headspace in the reactor, connection of tubes, etc.). The O2 concentration remained constant at ∼55 nM between the start of recording and the start of gassing. Consequently, it should be safe to assume that this was approximately the level of O2 in the reactor for the first 3 hours after filling (indicated by the gray line). The increase in O2 from 6–26 hours is due to leakage into the reactor or release of O2 from the PVC and rubber. Electronic errors have been removed. (PNG) [file pone.0037118.s001.png]

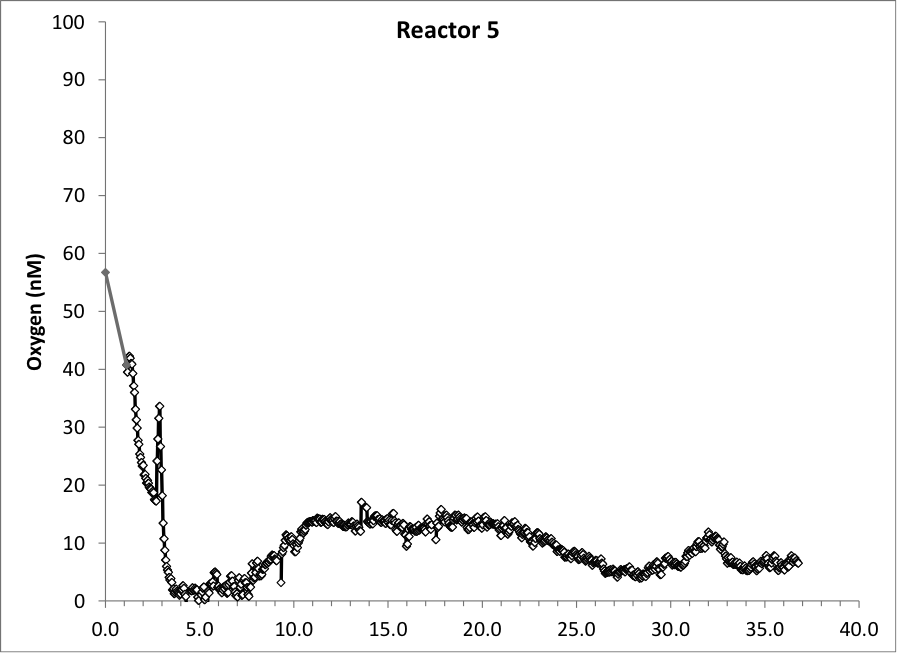

Supplement: Figure S2 — Dissolved O2 concentrations in the no amendment control bioreactor (#5) during Experiment B. STOX oxygen sensors began recording approximately 1 hour after bioreactor filling. Oxygen decreased from 40.7 nM to 17.5 over the first 1.5 hours ( = 15.5 nM h−1), potentially due to respiration. This bioreactor had been in use for several days and the release of O2 from PVC and rubber was smaller than in Experiment A above. Gassing with helium started at ∼2.7 hours, which created an increase in O2 due to the handling of the reactor. The best estimate of starting O2 concentration is 40.7+1.03×15.5 = 56.7 nM, as indicated by the grey line. Electronic errors have been removed. (PNG) [file pone.0037118.s002.png]
